# Supplementary material for: Reconciling Mining with the Conservation of Cave Biodiversity: A Quantitative Baseline to Help Establish Conservation Priorities
Source: PLoS One. 2016 Dec 20;11(12):e0168348. doi: 10.1371/journal.pone.0168348 (PMC5173368; doi:10.1371/journal.pone.0168348)
Supplement: S1 Dataset — (ZIP) [file pone.0168348.s002.zip › Taxa/Serra Norte/SN_2013/N4E/Lista N4E-33.pdf]

CAVIDADE N4E-0033

Classe	Ordem	Fam/Outros	Gên/Outros	Espécie	S-U	U-S	Zon.
Annelida	Oligochaeta			jovem	7		E/P/A
Arachnida	Acari	(Oribatida)		sp.8	X	X	P
Arachnida	Acari	Eupodidae		sp.1	X		P/A
Arachnida	Acari	Holothyrida		sp.		X	
Arachnida	Acari	Johnstonianidae		sp.68	X		P
Arachnida	Acari	Labidostommatidae		sp.1	X	X	E/P
Arachnida	Acari	Mesostigmata		jovem	X	X	P/A
Arachnida	Acari	Neothyridae	*Diplothyrus*	sp.1	X		P
Arachnida	Acari	Prostigmata		sp.49	X	X	P/A
Arachnida	Acari	Rhagidiidae		sp.20	X		P
Arachnida	Amblypygi	Charinidae	*Charinus*	sp.1	3	X	P/A
Arachnida	Amblypygi	Phrynidae	*Heterophrynus*	*longicornis*	24	X	P/A
Arachnida	Araneae	Barychelidae		jovem	1		P
Arachnida	Araneae	Caponiidae		sp.1	X		A
Arachnida	Araneae	Corinnidae		sp.1	1	1	P
Arachnida	Araneae	Ctenidae		jovem	6	1	P/A
Arachnida	Araneae	Dipluridae		jovem	3		
Arachnida	Araneae	Drymusidae		jovem	X		E
Arachnida	Araneae	Mysmenidae	*Mysmenopsis*	sp.1		X	
Arachnida	Araneae	Ochyroceratidae	*Ochyrocera*	sp.		X	
Arachnida	Araneae	Ochyroceratidae	*Ochyrocera*	sp.1	X		E/P/A
Arachnida	Araneae	Ochyroceratidae	*Ochyrocera*	sp.3	X		P
Arachnida	Araneae	Ochyroceratidae	*Speocera*	sp.1	X	X	E/P
Arachnida	Araneae	Ochyroceratidae	*Theotima*	sp.		X	
Arachnida	Araneae	Oonopidae		jovem	X		E
Arachnida	Araneae	Pholcidae	*Leptopholcus*	sp.1	X		P
Arachnida	Araneae	Pholcidae	*Mesabolivar*	aff. *cambridgei*	X		
Arachnida	Araneae	Pholcidae	*Mesabolivar*	*aurantiacus*	X		
Arachnida	Araneae	Pholcidae	*Mesabolivar*	sp.		X	
Arachnida	Araneae	Pholcidae	*Mesabolivar*	sp.1	X		P
Arachnida	Araneae	Pholcidae	Ninetinae	sp.1	X		P
Arachnida	Araneae	Salticidae		sp.7	X		P
Arachnida	Araneae	Scytodidae	*Scytodes*	sp.		X	
Arachnida	Araneae	Segestriidae		jovem		X	
Arachnida	Araneae	Theraphosidae	aff. *Holothele*	sp.1	6	X	
Arachnida	Araneae	Theridiidae	*Theridion*	sp.1	X		E
Arachnida	Araneae	Theridiosomatidae	*Plato*	sp.1	X	X	E/P/A
Arachnida	Opiliones	Escadabiidae		sp.2	X	X	P/A
Arachnida	Opiliones	Stygnidae	*Protimesius*	*laevis*	1	X	
Arachnida	Palpigradi	Eukoeneniidae	*Allokoenenia*	sp.1	X	X	P/A
Arachnida	Pseudoscorpiones	Chernetidae	*Spelaeochernes*	sp.1	X	X	P
Arachnida	Pseudoscorpiones	Chthoniidae		jovem	X	X	P
Arachnida	Scorpiones	Buthidae	*Ananteris*	sp.1	2		P
Chilopoda	Geophilomorpha	Mecistocephalidae	*Mecistocephalus*	sp.1	1	X	P
Chilopoda	Scolopendromorpha	Cryptopidae	*Cryptops*	jovem	1		E
Chilopoda	Scolopendromorpha	Scolopocryptopidae	*Dinocryptops*	*miersii*	2	X	P
Chilopoda	Scolopendromorpha	Scolopocryptopidae	*Newportia*	*lasia*		X	
Chilopoda	Scutigeromorpha	Pselliodidae	*Sphendononema*	*guildingii*	6	X	P
Diplopoda	Polydesmida	Chelodesmidae		sp.3	4	X	P
Diplopoda	Polydesmida	Fuhrmannodesmidae		sp.1	X		E/P/A
Diplopoda	Polydesmida	Pyrgodesmidae		sp.1	X	X	E/P/A
Diplopoda	Spirostreptida	Pseudonannolenidae	*Pseudonannolene*	jovem	2	X	P/A
Entognatha	Collembola	Cyphoderidae		sp.1	X		P
Entognatha	Collembola	Entomobryidae		sp.1	X		E

Entognatha	Collembola	Isotomidae	*Isotomiella*	*nummulifer*	X		E/P/A
Entognatha	Collembola	Paronellidae		sp.1	X		E/P
Entognatha	Collembola	Paronellidae		sp.4	X		P/A
Entognatha	Collembola	Sminthuroidea		sp.2	X	X	
Entognatha	Diplura	Campodeidae		sp.1	X		P
Entognatha	Diplura	Projapygidae		sp.1		X	
Insecta	Blattodea	Blattellidae		jovem	18	X	P
Insecta	Coleoptera	Carabidae	*Coarazuphium*	sp.n.1		X	A
Insecta	Coleoptera	Chrysomelidae		sp.13	X		E
Insecta	Coleoptera	Curculionidae	Scolytinae	sp.4	X		P
Insecta	Coleoptera	Staphylinidae		sp.34	X		P
Insecta	Coleoptera	Staphylinidae		sp.76	X		E
Insecta	Diptera	Chironomidae		sp.	X		P
Insecta	Diptera	Culicidae		sp.		X	
Insecta	Diptera	Dolichopodidae		sp.	X		P
Insecta	Diptera	Drosophilidae	*Drosophila*	*eleonorae*	X	X	E/P/A
Insecta	Diptera	Milichiidae		sp.	X		A
Insecta	Diptera	Phoridae	Phorinae	sp.	X		P
Insecta	Diptera	Psychodidae	Phlebotominae	sp.	X		P
Insecta	Diptera	Sciaridae		sp.	X		P
Insecta	Diptera	Tipulidae	Tipulinae	sp.	X	X	
Insecta	Heteroptera	Cydnidae		sp.	X	X	P/A
Insecta	Heteroptera	Dipsocoroidea		sp.	X		P
Insecta	Heteroptera	Nabidae	*Arachnocoris*	jovem	X		P
Insecta	Heteroptera	Reduviidae	Reduviinae	sp.	2		A
Insecta	Homoptera	Cixiidae		sp.7	X		P
Insecta	Hymenoptera	Diapriidae		sp.1	X		E
Insecta	Hymenoptera	Diapriidae		sp.2	X		A
Insecta	Hymenoptera	Formicidae	*Camponotus*	sp.		X	
Insecta	Hymenoptera	Formicidae	*Camponotus*	sp.1	X		P
Insecta	Hymenoptera	Formicidae	*Camponotus*	sp.2	X		P
Insecta	Hymenoptera	Formicidae	*Gnamptogenys*	*striatula*		X	
Insecta	Hymenoptera	Formicidae	*Hypoponera*	sp.1	X		P
Insecta	Hymenoptera	Formicidae	*Pachycondyla*	sp.2	X		P
Insecta	Hymenoptera	Formicidae	*Paratrechina*	sp.1	X		E
Insecta	Hymenoptera	Formicidae	*Paratrechina*	sp.2	X		E/P
Insecta	Hymenoptera	Formicidae	*Pheidole*	sp.		X	
Insecta	Hymenoptera	Formicidae	*Solenopsis*	sp.1	X		P
Insecta	Hymenoptera	Scelionidae		sp.		X	
Insecta	Isoptera	Termitidae		sp.		X	
Insecta	Lepidoptera	Noctuoidea		sp.5	1		P
Insecta	Lepidoptera	Tineoidea		jovem		X	
Insecta	Orthoptera	Gryllidae		sp.	X		E
Insecta	Orthoptera	Phalangopsidae	*Paraclodes*	sp.	4		E/P
Insecta	Orthoptera	Phalangopsidae	*Phalangopsis*	sp.	560	X	P/A
Insecta	Psocoptera	Epipsocidae	*Epipsocus*	sp.1	X		E
Insecta	Psocoptera	Epipsocidae	*Epipsocus*	sp.2	X		E/P
Insecta	Psocoptera			jovem	X	X	P
Gastropoda	Pulmonata	Bulimulidae	*Naesiotus*	sp.	X		E/P/A
Gastropoda	Pulmonata	Subulinidae	*Lamellaxis*	sp.		X	
Gastropoda	Pulmonata	Subulinidae	*Leptinaria*	sp.		X	
Gastropoda	Pulmonata	Systrophiidae	*Happia*	sp.	X	X	P
Malacostraca	Isopoda	Armadillidae		sp.1		X	
Malacostraca	Isopoda	Scleropactidae	*Circoniscus*	*buckupi*	X	X	P/A
Symphyla		Scutigerellidae	*Hanseniella*	sp.	X	X	
Amphibia	Anura	Strabomantidae	*Pristimantis*	cf. *fenestratus*	15	1	E/P
Mammalia	Chiroptera	Phyllostomidae	*Carollia*	*perspicillata*	20		P/A
Mammalia	Chiroptera	Phyllostomidae	*Glossophaga*	*soricina*	60		P/A

Mammalia	Chiroptera			sp.		100	E/P/A
Mammalia	Rodentia	Cricetidae	*Rhipidomys*	sp.	3		P
